# Generic and Specific Models for Volume Estimation in Forest and Savanna Phytophysiognomies in Brazilian Cerrado

**DOI:** 10.3390/plants13192769

**Published:** 2024-10-03

**Authors:** Yanara Ferreira de Souza, Eder Pereira Miguel, Adriano José Nogueira Lima, Álvaro Nogueira de Souza, Eraldo Aparecido Trondoli Matricardi, Alba Valéria Rezende, Joberto Veloso de Freitas, Hallefy Junio de Souza, Kennedy Nunes Oliveira, Maria de Fátima de Brito Lima, Leonardo Job Biali

**Affiliations:** 1 Department of Forest Science, Campus Darcy Ribeiro, University of Brasília (UnB), Brasília 70910-900, Brazil; yanarade@gmail.com (Y.F.d.S.); edermiguel@unb.br (E.P.M.); ansouza@unb.br (Á.N.d.S.); ematricardi@unb.br (E.A.T.M.); albavr@unb.br (A.V.R.); hallefyj.souza@gmail.com (H.J.d.S.); kennedynuneso@gmail.com (K.N.O.); ljbiali@unb.br (L.J.B.); 2 Tropical Forestry Department, National Institute for Amazon Research (INPA), Manaus 69067-375, Brazil; adriano@inpa.gov.br; 3 Faculty of Agricultural Sciences, Department of Forest Engineering, Federal University of Amazonas (UFAM), Manaus 69080-900, Brazil; jobertovf@gmail.com; 4 Forest Products Laboratory (LPF), Brazilian Forest Service (SFB), Brasília 70818-900, Brazil

**Keywords:** Cerrado biome, Forest Inventory, Dry Forest, Gallery Forest, Forest Savannah, Cerrado

## Abstract

The Cerrado has high plant and vertebrate diversity and is an important biome for conserving species and provisioning ecosystem services. Volume equations in this biome are scarce because of their size and physiognomic diversity. This study was conducted to develop specific volumetric models for the phytophysiognomies Gallery Forest, Dry Forest, Forest Savannah, and Savannah Woodland, a generic model and a model for Cerrado forest formation. Twelve 10 m × 10 m (100 m²) (National Forest Inventory) plots were used for each phytophysiognomy at different sites (regions) of the Federal District (FD) where trees had a diameter at breast height (DBH; 1.30 m) ≥5 cm in forest formations and a diameter at base height (Db; 0.30 m) ≥5 cm in savanna formations. Their diameters and heights were measured, they were cut and cubed, and the volume of each tree was obtained according to the Smalian methodology. Linear and nonlinear models were adjusted. Criteria for the selection of models were determined using correlation coefficients, the standard error of the estimates, and a graphical analysis of the residues. They were later validated by the chi-square test. The resultant models indicated that fit by specific phytophysiognomy was ideal; however, the generic and forest formation models exhibited similar performance to specific models and could be used in extensive areas of the Cerrado, where they represent a high potential for generalization. To further increase our understanding, similar research is recommended for the development of specific and generic models of the total volume in Cerrado areas.

## 1. Introduction

The biological complexity of tropical forests, associated with their loss of cover in recent decades, has increased the scientific demand for studies involving structural, floristic, and production data. Among the different types of tropical forests in the world, there are the forests of the Brazilian Cerrado biome. The Cerrado is the second largest biome in Latin America [1] and a provider of ecosystem services that include climate regulation and water supply to the richest and poorest regions of the country. The stability and functioning of neighboring ecosystems depend on the biological integrity of the Cerrado [2,3,4].

Recognizing the volumetric potential of this biome is essential because it will enable the composition of management plans with goals of production, conservation, and preservation [5]. Studies that attempt to estimate the volume of the Cerrado areas are scarce [6,7,8] because of the heterogeneous structure of the vegetation, such as in the shapes of trunks and crowns [9], and environmental variability [10].

The lack of volumetric models for native forests represents a gap in the knowledge about volume stocks in natural forest areas [11]. Volume prediction is related to independent variables collected in the field, such as diameter at breast height and total tree height [12], through equations.

The prediction of volume normally depends on equations, which are a tool to estimate volume from forest inventory data [13,14,15], through independent variables such as the diameter and total height of the individual [16,17,18,19]. These equations can be developed locally and are best suited to local species [18] or through generalized equations that apply to multiple tree species [20,21,22,23].

The use of mathematical models (indirect method) based on biometric data becomes a viable alternative for estimating tree volume [24]. Volumetric models are the most commonly used tools to estimate tree volume from forest inventory data. These models are derived from destructive methods; however, the number of trees felled is relatively small compared to the number of trees sampled in forest surveys.

In this context, the goals of this study were to (1) develop local equations to estimate the total volume of the phytophysiognomies Gallery Forest, Dry Forest, Forest Savannah, and Savannah Woodland of the Cerrado biome; (2) develop a generic volume equation for the Cerrado; (3) develop a volume equation for the forest formation set (Gallery Forest, Dry Forest, and Forest Savannah); and (4) evaluate whether the fit by specific physiognomy was ideal when compared to generic models of the Cerrado.

## 2. Results

### 2.1. Species Exclusivity

**Figure 1:**
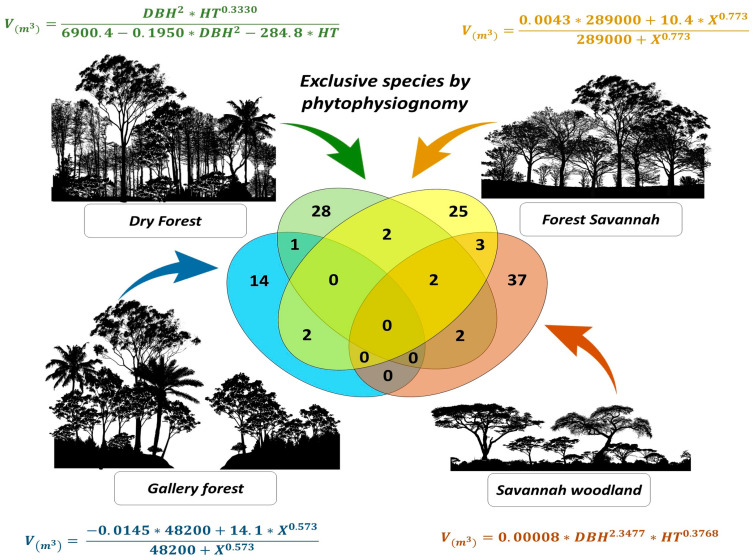
Venn diagram illustrating species sharing and exclusivity for different phytophysiognomies.

Regarding exclusive species, 14 were identified in Gallery Forest, 28 in Dry Forest, 25 in Forest Savannah, and 37 in Savannah Woodland (Figure 1). Two species were common in the three phytophysiognomies. Gallery Forest and Savannah Woodland did not share species, possibly because of differences in the environments.

### 2.2. Adjustment of Models to Estimate Total Volume

The models presented values for the correlation coefficient (r) equal to or greater than 0.91, except for model (1) for Savannah Woodland, the generic model, and the forest formation model. The standard errors of the estimate (Syx%) of the models exhibited values between 12.71% and 72.28%. Table 1 shows the results of the adjustment and statistical criteria used to select the best models. The model residues were normal (Shapiro–Wilk test).

**Figure 2:**
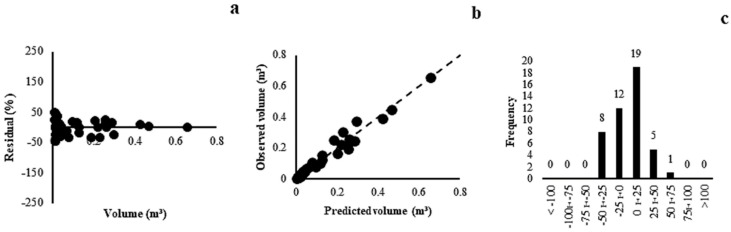
Residual dispersal (**a**), observed and predicted values (**b**) and distribution of error classes (**c**) for Gallery Forest model (6).

The models adjusted for the Gallery Forest presented correlation coefficients between 0.94 and 0.97 and standard errors of the estimate between 18.78% and 42.24%. Model 6 (r = 0.96, Syx = 23.74%) was superior in relation to the other models, with a uniform distribution of residues over the range of volume values (Figure 2a), estimated and observed volumes close to the slope line (Figure 2b), and a histogram of error frequencies at approximately ±25% (Figure 2c).

**Figure 3:**
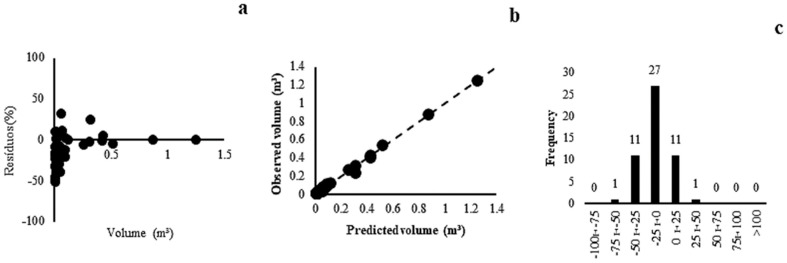
Residual dispersal (**a**), observed and predicted values (**b**) and distribution of error classes (**c**) for the Dry Forest model (3).

The models presented close correlation coefficients and standard errors of the estimate for Dry Forest, with a variation between 12.18% and 41.40%. Model (3) (r = 0.99, Syx = 12.71%) was selected as the most appropriate, with a tendency to overestimate the smallest volumes in the waste graph (Figure 3a). However, the accurate predictions of estimated and observed volumes (Figure 3b) and the concentration of residues in the central classes (Figure 3c) demonstrated the superiority of this model.

**Figure 4:**
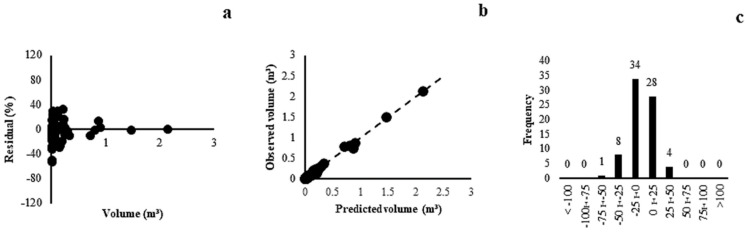
Residual dispersal (**a**), observed and predicted values (**b**) and distribution of error classes (**c**) for the model (6) of Forest Savannah.

In Forest Savannah, the models presented a correlation coefficient between 0.98 and 0.99, except for model (1). A high adjustment of the correlation coefficient indicated that most of the variance was explained by the models. Regarding the standard error of the estimate, there was variation between 14.58% and 25.44%. Model (6) was selected as the most appropriate (r = 0.99, Syx = 14.58%), with low data variability (Syx minor), with graphs of residues distributed evenly along the regression line (Figure 4a), volume values estimated and observed close to the 45° axis (Figure 4b), and a higher frequency of residues grouped in the class amplitude of ±25% (Figure 4c).

**Figure 5:**
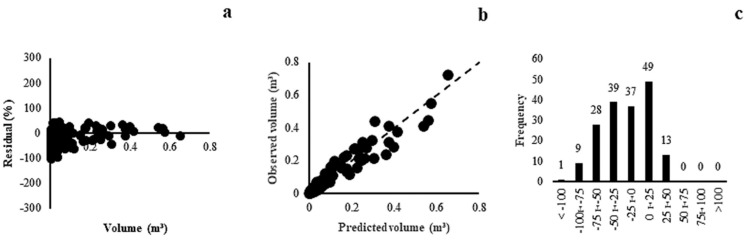
Residual dispersal (**a**), observed and predicted values (**b**) and distribution of error classes (**c**) for the model (4) of Savannah Woodland.

In the Savannah Woodland phytophysiognomy, the correlation coefficient showed close values. The standard errors of the estimate varied between 41.69% and 49.59%. Model (4) showed better adjustment results (r = 0.93, Syx = 41.69%), which corroborated the uniform residual distribution (Figure 5a), because these values were distributed close to the 45° slope (Figure 5b), and the histogram of the frequency of errors occurred in the central classes (Figure 5c).

**Figure 6:**
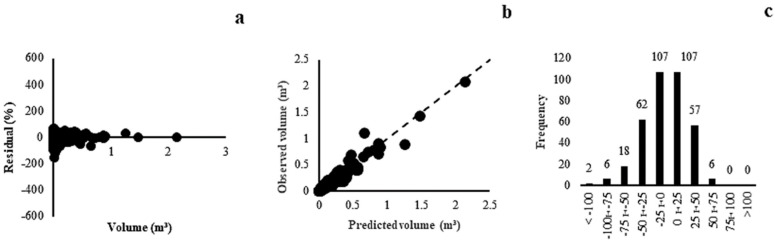
Residual dispersal (**a**), observed and predicted values (**b**) and distribution of error classes (**c**) for model (6) of generic model.

For the generic model, the correlation coefficient ranged from 0.93 to 0.97 and the standard error of the estimate was between 45.53% and 51.65%. Model (6) (r = 0.95, Syx = 46.23%) presented the best fit and precision statistics; however, these statistics were inferior to the specific models because of the high variability of the data attributable to different phytophysiognomies and the larger number of species than those in the other models in this study. The selected model showed a homogeneous distribution along the waste line (Figure 6a), with estimated volume values observed close to the slope of the line (Figure 6b) and a greater concentration of residues within ±25% (Figure 6c).

**Figure 7:**
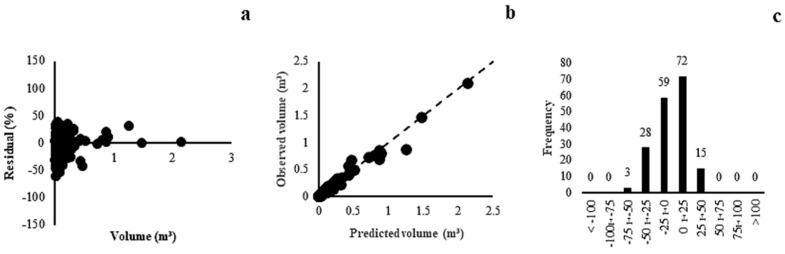
Residual dispersal (**a**), observed and predicted values (**b**) and distribution of error classes (**c**) for the model (4) of Forest Formation.

In forest formation (Gallery Forest, Dry Forest, and Forest Savannah), the correlation coefficient varied between 0.95 to 0.98 and the standard error of the estimate was between 34.08% and 42.24%. Model (4) (r = 0.95, Syx = 34.08%) showed a lower tendency to overestimate and underestimate the graphical analysis of residues, with errors within ±60% (Figure 7a), estimated and observed volume values close to the slope line (Figure 7b), and a frequency of error classes within ±25% (Figure 7c), which reinforced the selection of this model. Models of generic volume and forest formation were new for the Cerrado biome. Models of this magnitude have been developed in only a few Brazilian states [22,23,24].

### 2.3. Validation of Volume Models

According to the results (Table 2), the chi-square test did not show statistical differences between the actual values of volume and those estimated by the models, which proved the reliability of the estimates generated by the selected models. The Kruskal–Wallis test (Table 3) indicated that the generic model and the forest formation model in relation to the specific models did not present a significant difference between the groups, which corroborated the reliable estimates for both models. However, as shown in the Venn diagram (Figure 1), most species were unique to each phytophysiognomy, strengthening the preference for specific models.

The aggregate difference (Table 3) showed some negative values for the generic model and the forest formation model. This result showed a tendency of these models to overestimate volume when used in specific regions. Most of the specific models, on the other hand, showed the inverse behavior, with positive values, which had a tendency to underestimate volumes; however, the values were close to the real ones.

## 3. Discussion

Through the Venn diagram, it was possible to view exclusive species, that is, those that occurred in only one phytophysiognomy. Phytophysiognomies that were closer to one another were also observed. Thus, the use of specific models influenced the volume estimate because most species were exclusive to the different types of Cerrado and no species were common to the four types studied.

Owing to the significant structural differences between phytophysiognomies, indicating that individuals with denser vegetation types invested more in upward growth than in diameter [25], the use of generic models may not detect this variation and overestimate or underestimate the volumes in the different phytophysiognomies of the Cerrado.

The residual plots, observed versus predicted, and frequency histograms were used to support the choice of models. Graphical interpretation alternatives, such as these, are necessary to the quality of the fit [26,27] and the choice of a model, considering that trend errors may occur and not be noticed by other statistics [7].

Regarding the phytophysiognomy of Gallery Forest, few models in the literature have been described related to volumetry [28]. The scarcity of works on this phytophysiognomy makes it difficult to compare the selected model with others in different locations. This is because of the substantial heterogeneity of the Cerrado, which often implies costly and bureaucratic volume estimates [8,15]. More volumetric studies are needed in these areas because the Brazilian legislation, according to the Forest Code, classifies this phytophysiognomy as a permanent preservation area (APP), being highly sought after for building condominiums, which makes environmental licensing mandatory for this type of enterprise.

In a study on Dry Forest, the adjustment statistics for the standard error of the estimate varied between 23.54% and 53.25% [22]. In general, model (3) had Syx values higher than those described in the literature, which could be explained by the specific models tested, together with the characteristics of the database.

The precision measurements of model (6) of the Forest Savannah were considered satisfactory and superior to previous studies on this phytophysiognomy [22,29,30]. The superiority of this model implied that specific models are preferable for volume estimation [31]. Although there was superiority in relation to this model chosen in this physiognomy, determining other methods for obtaining the volume is important. For example, in studies with artificial neural networks for modeling the volume of wood in Forest Savannah areas, more statistics of adjustments and higher precision were found than in the present study [7].

Model (4) for Savannah Woodland presented lower precision measures than those from previous studies estimating volume in this phytophysiognomy [5,6,9,32]. However, the selected model was satisfactory because of the natural variability of the vegetation structure, as already discussed by some authors [9,22]. The high variability of Savannah Woodland vegetation [15] reinforced the importance of considering different methods for estimating volume [8].

Efforts are needed to develop generic models in this biome that represent the forest and savanna vegetation of the Cerrado as a whole. Its use has some limitations for the effective support of forest management [33]; however, the generic models developed in this study included many individuals and could be used in large areas of the Cerrado where volume estimates could be generalized.

Volume models for natural forests at local or geographic scales are poorly studied [23] and highly recommended compared to generic models [33]. Volume estimates must be calculated according to the type of forest, respecting the composition and structure of species [23].

Another important factor in studies of generic and local models that hinders the development of new models is that the selection procedures do not consider the quality of the adjustments, the statistics associated with the model, the sampling design, and the information regarding the uncertainty of its parameters [23,26,34], and this affects volume modeling.

The choice of a model depends on the precision requirements, the degree of generality [35], and information from the study area. Generic models should prioritize larger samples [23] and be conducted in areas with a lack of local models because local models are recommended in regions of local inventories [36], which present floristic heterogeneity [23] in Cerrado phytophysiognomies.

The models developed in this study could be of great importance for understanding the carbon cycle in the Cerrado areas and for the accuracy of the greenhouse gas inventories in Brazil. The availability of models has been a limitation in assessing forest volume [37,38] and the development of specific and generic models will contribute to the improvement of estimates of this variable. Considering the cost of improving volume estimates for the Cerrado, further studies with forecasting techniques such as nonlinear regression, satellite data, and artificial intelligence techniques are needed [7,8,16,39,40] to understand changes in the biome and stimulate the sustainable use of forests and the carbon credit market [14,41,42].

Other forms of non-destructive sampling, such as LiDAR, are essential in future studies for collecting dendrometric data. This type of sampling guarantees larger sample sizes. It can be conducted systematically in the field and becomes more viable in protected areas [43,44], such as in Gallery Forests.

Thus, the choice of a model depends on the desired precision. In general, in large regions of the Cerrado where no specific models are available, the generic model and the forest formation model developed in this study offer an alternative to specific models. These models share different species, which increases the variability of the data in relation to specific models [23] and contributes to improving volume estimates because some specific models are developed from small samples, resulting in skewed estimates [45].

## 4. Materials and Methods

### 4.1. Location and Characterization of the Study Area

This study was conducted in the Federal District in the central region of Brazil for forest phytophysiognomies (Gallery Forest, Dry Forest, and Forest Savannah) and savannas (Savannah Woodland) of the Cerrado biome. According to the MapBiomas platform (https://mapbiomas.org/estatisticas, accessed on 14 May 2024), in 2019, the Cerrado attained an area of 14.7% of forest formations and 30.2% of savanna formations.

The Cerrado has a tropical climate with two well-defined seasons: the rainy season from October to March and the dry season from April to September. The average annual rainfall is 1500 mm and average temperatures vary between 22 °C and 27 °C [46].

### 4.2. Data Collection

**Figure 8:**
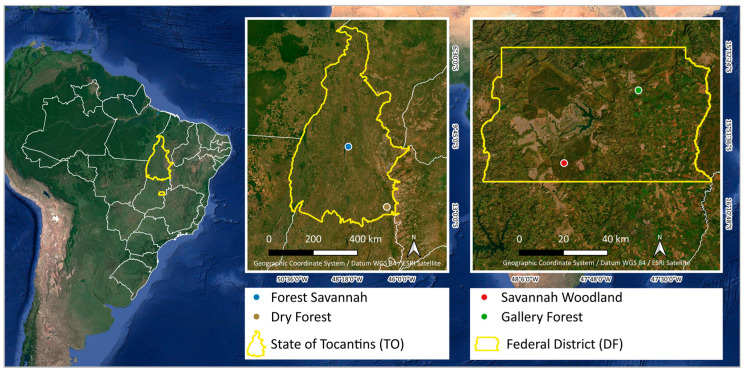
Location of sampling points of the different phytophysiognomies, Brazil.

The forest inventory conducted in the study area followed a random sampling process, with 12 plots of 10 m × 10 m (100 m²) at four sites (regions) including Cerrado forest and savanna typologies (Figure 8).

In the plots, all trees with a diameter equal to or greater than 5 cm, at 1.3 m from the soil (diameter at breast height; DBH), for forest formations, and those with a diameter equal to or greater than 5 cm, at the base (diameter at base; Db), for savanna formation were sampled.

The dataset consisted of 17 to 44 species (380 individuals in total—Table 4), distributed between forest formations (Gallery Forest, Dry Forest, and Forest Savannah) and savanna formation (Savannah Woodland).

To analyze the exclusivity of species among phytophysiognomies (Gallery Forest, Dry Forest, Forest Savannah, Savannah Woodland), a Venn diagram was constructed using InteractiVenn [47]. This diagram was used to visualize the interactions between the various datasets [48] based on the presence or absence of species.

Subsequently, the trees in each plot were cut, their total heights were measured, they were sequentially cubed, and the volume of each individual was obtained.

The total volume was determined through rigorous cubing, in which the shaft of each individual was measured in sections of variable length by using the formula of Smalian [49]:

(1) $$V=\frac{\mathrm{AS}1+\mathrm{AS}2}{2}\cdot \;L$$

Here, V is the section volume measured in m³, AS1 and AS2 are the sectional areas obtained at the ends of each section (m²), and L is the section length (m). The total volume of each individual was obtained as the sum of the volumes of each section.

### 4.3. Adjustment of Volumetric Models and Statistical Analysis

All data underwent a preliminary analysis to ensure a normal distribution using the Shapiro–Wilk test (α = 0.05) and removal of outliers when detected by the Dixon Q test. A set of specific models for each phytophysiognomy, generic model, and forest formation model were developed. We selected the six models that exhibited the best performance (Table 5) for volume estimation, referring to Gallery Forest, Dry Forest, Forest Savannah, and Savannah Woodland for the generic dataset (data comprising all studied vegetation types) and a set of forest formations that corresponded to the three Cerrado phytophysiognomies (Gallery Forest, Dry Forest, and Forest Savannah).

The volume prediction was performed using tree measurement data: DBH, Db, and HT (total height of the tree). Some of the cubed trees (80%) were used to make the adjustments, and consequently, 20% were randomly assigned to perform model validation, using the chi-square test.

Adjustments of linear and nonlinear models were performed using the Statistica^®^ 12 program (Statsoft Inc., version 12, Tulsa, OK, USA). The linear regression model was adjusted by the ordinary least squares method (OLS) and the nonlinear models by the Levenberg–Marquardt (LM) method [50,51]. The CurveExpert^®^ 1.4 program (Hyams Development, Boston, MA, USA) was also used to adjust the sigmoidal model.

### 4.4. Model Selection Criteria

The selection of models to estimate volume was based on the following adjustment and precision criteria: correlation coefficient (r), standard error of the estimate in percentage (Syx%), and verification of homoscedasticity and normality through the graphic analysis of the residues [52]. The observed versus predicted graph [53] and the frequency histogram in relative error classes [27] were also used to select the best model. The determination coefficient was replaced by the correlation coefficient (r) for the nonlinear models.

(2) $$Syx\left(\%\right)=\frac{Syxabsolute}{\bar{Y}}\cdot 100$$

(3) $$\mathrm{Syx}\;\mathrm{absolute}=\sqrt{\frac{{\left(Yi-Ye\right)}^{2}}{n-P}}$$

Here, Syx(%) is standard error of the estimate in percentage, Syx absolute is standard error of the estimate absolute, $\bar{Y}$ is average volume of the observed trees, Yi is volume observed, Ye is volume estimate, n is number of observations, and P is number of coefficients of the mathematical model.

### 4.5. Validation of Adjusted Models

The selected models were submitted to a validation test to assess their efficiency. For the test, 20% of the total number of individuals was used for each phytophysiognomy. The chi-square test was used to validate the results.

The assumption of normality of residues in the 20% of individuals used for validation was not met; thus, the Kruskal–Wallis non-parametric test (α = 0.05) was performed to verify statistical differences between the generic model, forest formation model, and specific models.

An accuracy test was also conducted using the aggregated difference method [7], shown in Equation (2), which indicated the existence of biases. This test was applied to the specific, generic, and forestry models against the actual value of total volume.

(4) $$DA=\frac{\Sigma Yobs-\Sigma Yest}{\Sigma Yobs}\cdot 100$$

Here, DA is the aggregate difference in percentage, ΣY obs is the sum of the actual volume values (m³), and ΣY est is the sum of the values of the volume estimated by the model (m³).

## 5. Conclusions

The following conclusions were obtained: (1) the results of our research will aid in estimating and elucidating the total volume in the forest and savanna phytophysiognomies of the Cerrado biome; (2) in large areas of the Cerrado where they have a high potential for generalization (different phytophysiognomies), the generic and forestry models could be used because they present similar performance to specific models; (3) the results of this study will contribute to helping researchers, environmental agencies, and professionals in the environmental field in estimating the total volume of the main forest and savanna phytophysiognomies of the Cerrado biome; and (4) considering the diversity of the Cerrado, it is recommended that similar research on the development of new specific and generic models of total volume should be conducted, including the other savannas and countryside phytophysiognomies.

## Figures and Tables

**Table 1:** Estimates of parameters and precision measures of the models developed to estimate total volume for phytophysiognomies in the Cerrado biome, Brazil. r: correlation coefficient; Syx%: standard error of the estimate.

	Model	Parameters				r	Syx (%)
		$\beta_{0}$	$\beta_{1}$	$\beta_{2}$	$\beta_{3}$		
Gallery Forest	1	0.000019	0.832375	-	-	0.93	26.08
	2	0.000075	0.913827	1.59674	0.428156	0.95	25.75
	3	−0.8557	831.0178	0.0800	−48.0847	0.97	18.78
	4	0.0001	1.8276	0.5967	-	0.96	24.18
	5	0.0120	0.00003	-	-	0.95	27.96
	6	−0.0145	48,200	14.1	0.573	0.96	23.74
Dry Forest	1	0.000015	0.9021676	-	-	0.99	14.18
	2	0.000004	1.041667	2.493624	0.328918	0.99	14.05
	3	0.3330	6900.4590	−0.1950	−284.8510	0.99	12.71
	4	0.00001	2.0833	1.4936	-	0.99	17.09
	5	−0.0084	0.00006	-	-	0.98	29.58
	6	0.0043	1,260,000	31.6000	0.8030	0.96	41.40
Forest Savannah	1	0.00004	0.7796656	-	-	0.96	25.44
	2	0.000026	1.054775	1.655038	0.280647	0.99	17.18
	3	0.5900	6982.462	−0.1970	−35.9500	0.99	16.46
	4	0.00009	2.1095	0.6550	-	0.99	15.27
	5	0.0008	0.00005	-	-	0.98	22.57
	6	0.0043	289,000	10.4000	0.7730	0.99	14.58
Savannah Woodland	1	0.0003	0.656207	-	-	0.94	47.90
	2	0.00005	1.173887	1.376810	0.598396	0.96	49.59
	3	1.3010	6053.714	−6.8880	3134.1960	0.93	42.10
	4	0.00008	2.3477	0.3768	-	0.93	41.69
	5	0.0059	0.00006	-	-	0.91	47.69
	6	0.0014	51,100	2.0900	0.7880	0.93	42.87
Generic	1	0.00012	0.7102178	-	-	0.93	48.79
	2	0.000078	1.066592	1.611996	0.840370	0.97	49.05
	3	0.3680	4975.5890	−0.2010	−75.7320	0.95	45.53
	4	0.00009	2.1331	0.6119	-	0.95	45.87
	5	0.0082	0.00005	-	-	0.93	51.65
	6	−0.0020	2,150,000	207	0.6890	0.95	46.23
Forest Formation	1	0.00004	0.7800377	-	-	0.95	39.43
	2	0.000021	1.078534	1.753315	0.352431	0.94	42.24
	3	0.6380	9218.8480	−0.4137	−78.3180	0.95	35.11
	4	0.00005	2.1250	0.7874	-	0.95	34.08
	5	−0.0007	0.00005	-	-	0.95	37.04
	6	0.00006	1,370,000	61.2000	1.0100	0.94	39.44

**Table 2:** Statistics of the chi-square test (χ²) at 95% probability in the different typologies analyzed for volumetric estimation in Cerrado, Brazil.

Phytophysiognomies	χ² Calculated	χ² Tabulated
Gallery Forest	0.020	2.73
Dry Forest	0.002	3.33
Forest Savannah	0.080	6.57
Savannah Woodland	0.200	21.66
Generic	0.400	51.74
Forest Formation	0.140	22.47

Note: there were 8 degrees of freedom in Gallery Forest, 9 degrees of freedom in Dry Forest, 14 degrees of freedom in Forest Savannah, 34 degrees of freedom in Savannah Woodland, 72 degrees of freedom in the generic model, and 35 degrees of freedom in the forest formation model.

**Table 3:** Statistics of the aggregated difference (DA%) of the generic model, forest formation model, and specific models and the Kruskal–Wallis test (H) for the total volume of vegetation types in the Cerrado, Brazil. REAL: real values; ESP: specific model; GEN: generic model; FLOR: forest model; ns: not significant at the 95% probability level.

	Phytophysiognomies	Method	DA (%)	H
Generic	Gallery Forest	REAL × ESP REAL × GEN	−2.20 −35.63	1.79 ^ns^
	Dry Forest	REAL × ESP REAL × GEN	0.24 −1.53	0.02 ^ns^
	Forest Savannah	REAL × ESP REAL × GEN	4.31 4.99	0.02 ^ns^
	Savannah Woodland	REAL × ESP REAL × GEN	−3.91 10.94	0.86 ^ns^
	Forest Formation	REAL × ESP REAL × GEN	−1.70 −6.42	0.23 ^ns^
Forest Formation	Gallery Forest	REAL × ESP REAL × FLOR	−2.20 −21.48	1.17 ^ns^
	Dry Forest	REAL × ESP REAL × FLOR	0.24 9.28	0.44 ^ns^
	Forest Savannah	REAL × ESP REAL × FLOR	4.31 10.57	0.12 ^ns^

**Table 4:** Information from the database used in each phytophysiognomy. Quant. of trees: number of trees sampled in each phytophysiognomy; D medium: mean diameter (cm); D min: minimum diameter (cm); D max: maximum diameter (cm); H medium: average height (m); H min: minimum height (m); H max: maximum height (m); quant. of species: number of species in each phytophysiognomy.

Typology	Quant. of Trees	D Medium	D Min	D Max	H Medium	H Min	H Max	Quant. of Species
Gallery Forest	50	13.75	5.57	37.36	10.27	3.45	15.20	17
Dry Forest	58	10.94	5.00	39.00	7.04	3.19	15.70	35
Forest Savannah	80	12.52	5.30	49.00	10.26	3.80	24.50	34
Savannah Woodland	192	11.28	5.00	32.30	3.88	1.00	11.10	44

**Table 5:** Adjusted models to estimate the total volume for forest and savanna formations in the Cerrado biome, Brazil. V: total volume of the tree (m³); DBH: diameter at 1.30 m from the ground for forest formations (cm); Db: diameter 0.30 m from the ground for savanna formations (cm); HT: total height of the tree (m); X: DBH^3^·HT; ε: error associated with the model.

Models		Families
$V=\beta 0\cdot {{(DBH}^{2}\cdot {HT}^{2})}^{\beta 1}+\varepsilon$	(1)	Non-linear
$V=\beta 0\cdot ({DBH^{2})}^{\beta 1}\cdot \frac{{HT}^{\beta 2}}{\beta 3.HT}+\varepsilon$	(2)	Non-linear
$V=\frac{DBH^{2}\cdot {HT}^{\beta 0}}{\beta 1+\beta 2\cdot DBH^{2}+\beta 3\cdot HT}+\varepsilon$	(3)	Non-linear
$V=\beta 0\cdot {DBH}^{\beta 1}\cdot {HT}^{\beta 2}+\varepsilon$	(4)	Non-linear
$V=\beta 0+\beta 1\cdot DBH^{2}\cdot HT+\varepsilon$	(5)	Linear
$V=\frac{\beta 0\cdot \beta 1+\beta 2\cdot X^{\beta 3}}{\beta 1+X^{\beta 3}}+\varepsilon$	(6)	Non-linear

## Data Availability

Data are contained within the article.
